# Supplementary material for: Altered microRNA Transcriptome in Cultured Human Liver Cells upon Infection with Ebola Virus
Source: Int J Mol Sci. 2021 Apr 6;22(7):3792. doi: 10.3390/ijms22073792 (PMC8038836; doi:10.3390/ijms22073792)
Supplement: Supplementary file 1 [file ijms-22-03792-s001.zip › Supplementary_File/C_ GO_Analysis_Results/16-30nt_go_Reston-96h-Huh7_vs_Control-96h-Huh7_up.mature_mirna_targets/MF_Pvalue_tree.pdf]

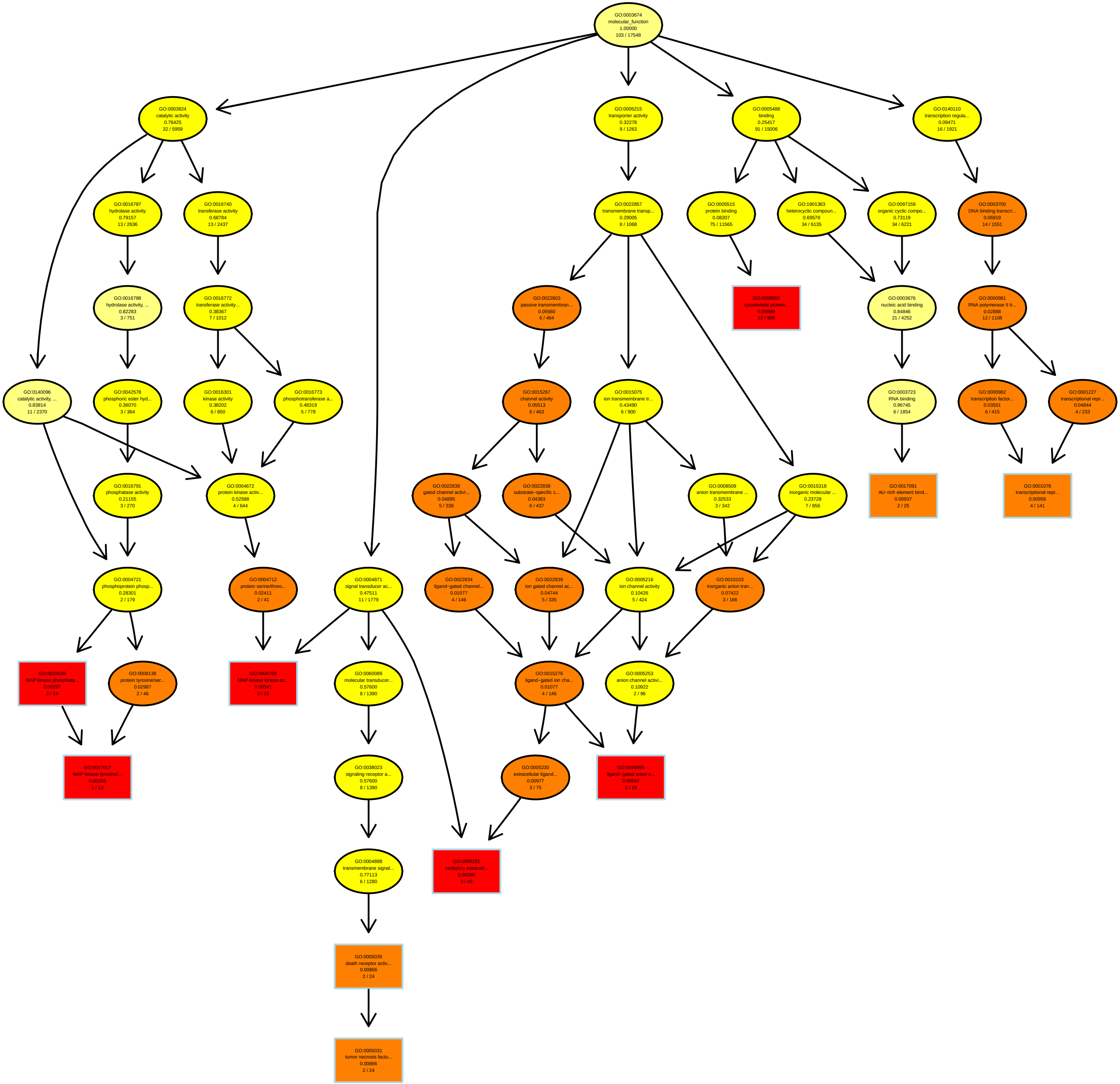

GO:0003674 molecular_function 1.00000 103 / 17548
GO:0003824 catalytic activity 0.76425 32 / 5959
GO:0005215 transporter activity 0.32278 9 / 1263
GO:0005488 binding 0.25417 91 / 15006
GO:0140110 transcription regula... 0.09471 16 / 1921
GO:0016787 hydrolase activity 0.79157 13 / 2636
GO:0016740 transferase activity 0.68784 13 / 2437
GO:0022857 transmembrane transp... 0.29005 8 / 1068
GO:0005515 protein binding 0.08207 75 / 11565
GO:1901363 heterocyclic compoun... 0.69579 34 / 6135
GO:0097159 organic cyclic compo... 0.73119 34 / 6221
GO:0003700 DNA binding transcri... 0.06919 14 / 1551
GO:0016788 hydrolase activity, ... 0.82283 3 / 751
GO:0016772 transferase activity... 0.38367 7 / 1012
GO:0022803 passive transmembran... 0.05560 6 / 464
GO:0008092 cytoskeletal protein... 0.00689 12 / 908
GO:0003676 nucleic acid binding 0.84846 21 / 4252
GO:0000981 RNA polymerase II tr... 0.02888 12 / 1108
GO:0140096 catalytic activity, ... 0.83814 11 / 2370
GO:0042578 phosphoric ester hyd... 0.36070 3 / 364
GO:0016301 kinase activity 0.38202 6 / 850
GO:0016773 phosphotransferase a... 0.48319 5 / 778
GO:0015267 channel activity 0.05513 6 / 463
GO:0015075 ion transmembrane tr... 0.43490 6 / 900
GO:0003723 RNA binding 0.96745 6 / 1854
GO:0000982 transcription factor... 0.03551 6 / 415
GO:0001227 transcriptional repr... 0.04844 4 / 233
GO:0016791 phosphatase activity 0.21155 3 / 270
GO:0004672 protein kinase activ... 0.52588 4 / 644
GO:0022836 gated channel activi... 0.04895 5 / 338
GO:0022838 substrate-specific c... 0.04383 6 / 437
GO:0008509 anion transmembrane ... 0.32533 3 / 342
GO:0015318 inorganic molecular ... 0.23728 7 / 856
GO:0017091 AU-rich element bind... 0.00937 2 / 25
GO:0001078 transcriptional repr... 0.00956 4 / 141
GO:0004721 phosphoprotein phosp... 0.28301 2 / 179
GO:0004712 protein serine/threo... 0.02411 2 / 41
GO:0004871 signal transducer ac... 0.47511 11 / 1779
GO:0022834 ligand-gated channel... 0.01077 4 / 146
GO:0022839 ion gated channel ac... 0.04744 5 / 335
GO:0005216 ion channel activity 0.10426 5 / 424
GO:0015103 inorganic anion tran... 0.07422 3 / 166
GO:0033549 MAP kinase phosphata... 0.00297 2 / 14
GO:0008138 protein tyrosine/ser... 0.02987 2 / 46
GO:0004708 MAP kinase kinase ac... 0.00341 2 / 15
GO:0060089 molecular transducer... 0.57600 8 / 1390
GO:0015276 ligand-gated ion cha... 0.01077 4 / 146
GO:0005253 anion channel activi... 0.10922 2 / 96
GO:0017017 MAP kinase tyrosine/... 0.00255 2 / 13
GO:0038023 signaling receptor a... 0.57600 8 / 1390
GO:0005230 extracellular ligand... 0.00977 3 / 75
GO:0099095 ligand-gated anion c... 0.00547 2 / 19
GO:0004888 transmembrane signal... 0.77113 6 / 1280
GO:0005231 excitatory extracell... 0.00280 3 / 48
GO:0005035 death receptor activ... 0.00866 2 / 24
GO:0005031 tumor necrosis facto... 0.00866 2 / 24
